# Assessment of platelets morphological changes and serum butyrylcholinesterase activity in children with diabetic ketoacidosis: a case control study

**DOI:** 10.1186/s12902-017-0174-6

**Published:** 2017-04-04

**Authors:** Suzan Omar Mousa, Samira Zein Sayed, Mahmoud Mohammed Moussa, Ahmed Hamdy Hassan

**Affiliations:** 1 Department of Pediatrics, Faculty of Medicine, Minia University, El-Minya, Egypt; 2 Department of Clinical Pathology, Faculty of Medicine, Minia University, El-Minya, Egypt

**Keywords:** Mean platelet volume, Platelet distribution width, Serum butyrylcholinesterase activity, Diabetic ketoacidosis

## Abstract

**Background:**

Many studies indicated that mean platelet volume (MPV) and platelet distribution width (PDW) may be valuable in the diagnosis and management of clinical disorders; also, serum butyrylcholinesterase activity (BChE) was suggested to be linked to systemic inflammation and oxidative stress. Limited studies measured these readily available markers in children with diabetic ketoacidosis (DKA). Our objectives were to measure MPV, PDW and BChE in children with DKA; and to assess if any of these markers reflects the severity of DKA.

**Methods:**

Our study included: 30 children with DKA (DKA group), 30 diabetic children (Non-DKA group) and 30 apparently healthy children (control group). MPV, PDW and BChE were measured in all children. Additional blood samples were withdrawn from the DKA group to assess these markers at discharge from hospital.

**Results:**

MPV, PDW and BChE were significantly altered in the DKA group than the other two groups; and their levels improved significantly at discharge of the DKA group (*p* < 0.05). The three markers were found to equally to predict the presence of DKA, but MPV was the most suitable risk marker for DKA diagnosis (OR = 4.251, CI 95% =1.463–12.351, *p* = 0.003). Regarding their relation with DKA severity, they did not correlate significantly with arterial PH or serum HCO3^-^ (*p* > 0.05).

**Conclusion:**

DKA in children is associated with changes in MPV, PDW and BChE activity, which improve after resolution of the condition. Elevated MPV can be a suitable risk marker for DKA. None of the studied markers correlated with the severity of DKA.

## Background

Diabetic ketoacidosis (DKA) represents a profound insulin deficient state leading to hyperglycemia (>200 mg/dl) and acidosis (serum pH < 7.3, HCO3 < 15 mEq/L), along with accumulation of keto-acids in the blood, dehydration, electrolyte loss, and hyperosmolality [1]. This severe insulin deficiency can occur in previously undiagnosed type 1 diabetes mellitus and when patients on treatment do not take insulin [2].

DKA is associated with the non-infectious form of the systemic inflammatory response and an increase in oxidative stress [3]. DKA-induced inflammation has been a recent focus of investigation [4]. Immediate diagnosis and early treatment of DKA are required in order to reverse this inflammatory state.

Understanding the role of platelets in a variety of thrombotic and inflammatory disorders has substantially improved owing to the recent advances in the quantification of laboratory markers of platelet function [5]. Platelet activation leads to changes in platelet shape with increase in platelet swelling leading to an increase in mean platelet volume (MPV) and platelet distribution width (PDW) [6]. The automated cell counter provides MPV and PDW on each whole blood sample that is processed, which makes possible the study of platelet size in a great variety of clinical conditions [7]. This is something clinicians should make good use of.

Butyrylcholinesterase (BChE), also known as pseudocholinesterase, is produced in the liver and found primarily in blood plasma [8]. Assessing serum BChE activity is the most commonly used diagnostic biomarker of acute organophosphorus poisoning [9].

Recently, serum BChE activity was suggested to serve as a marker for low-grade systemic inflammation [10] and oxidative stress [11] in some clinical conditions. Decreased pseudocholinesterase levels were found in: acute infection, chronic malnutrition, heart attack, liver damage, metastasis, obstructive jaundice, poisoning from organophosphates, inflammation that accompanies some diseases, pregnancy, and use of birth control pills [12]. The activity of BChE was found to be significantly altered in both type 1 and type 2 diabetics [13].

The aim of our study was to answer the following questions: Does DKA in children affect MPV, PDW and serum BChE activity? If yes, is there any correlation between these markers and DKA severity? Lastly, can any of these markers be used for DKA diagnosis?

## Methods

### Subjects

This case-control study was carried out at Minia University Children hospital over the period from June 2015 to January 2016. The study included: (1) DKA group: 30 children admitted to PICU with DKA according to NICE guidelines, 2015 [14]. (2) Non-DKA group: 30 children already diagnosed as having type 1 diabetes mellitus according to ADA, 2010 criteria [15], they were chosen during routine follow up visits to pediatric endocrinology outpatient clinic. (3) Control group: 30 apparently healthy children age and sex matched with the previous two groups.

Children suffering from the following were excluded from our study: heart failure, hematologic diseases, acute or chronic infections or liver disease. Also, children taking anticoagulants or steroids were excluded. All children underwent diabetes-focused physical examination including assessment of anthropometric measurements, vital signs (temperature, heart rate, respiratory rate, blood pressure), limited vascular and neurologic examinations. Other systems were assessed when indicated by the patient’s clinical situation.

### Methods

In the control and non-DKA groups, blood samples were collected after an overnight fast. In the DKA group, the blood and urine samples were taken at admission to the pediatric intensive care (PICU) and another blood sample at discharge from the hospital. Routine chemistry tests including plasma glucose, BUN, creatinine were performed using fully automated clinical chemistry analyzer Konelab 20i (Thermo Scientific, Finland). Blood samples for ABG were collected in heparin tubes and were determined by ABL90 FLEX blood gas analyzer (Radiometer Medical Apps, Denmark). Blood samples for HbA1c, platelet counts and the determination of MPV and PDW were collected in tubes with EDTA. The HbA1c was determined by kits supplied by (Stanbio Laboratory, Boerne, Texas). The platelet parameters were determined with the automated hematology cell counter Mindray BC-3600 (Mindray, Shenzhen, China). The activity of BChE was measured by an enzymatic method using a commercial kit supplied by (Biodiagnostics, Egypt).

### Statistical methods

The collected data were statistically analyzed using statistical package for social sciences (SPSS) program for windows version 20. Quantitative results were presented as mean ± standard deviation (SD) while qualitative data were presented by frequency distribution as percentage (%). Graphics were done by Excel Microsoft Office 2010. Student Newman–Keuls ANOVA was utilized for comparative analysis among the three groups, Chi square test, was used to compare between proportions, and Paired t-tests were utilized to determine the significance of changes in the DKA patients between the admission and the discharge. Receiver operating characteristic (ROC) curve analysis was performed using MedCalc_version 12.1.4.0. to determine: the optimal cut-off values and the diagnostic performance of the variable, the diagnostic sensitivity and specificity, and comparison of sensitivity and specificity for B.Ch.E. activity, MPV, PDW (z-statistics). Multiple logistic analyses were used to assess the risk markers for the diagnosis of DKA. The probability of error less than 0.05 was used as a cutoff point for all statistically significant tests. Correlation was performed by using Pearson correlation coefficient (r).

## Results

### The studied children

Detailed demographic and laboratory data of the three groups are presented in Table 1. Plasma glucose and glycated HbA1c were significantly higher in the DKA group than in the other two groups (*p* < 0.001 in both); and they were significantly higher in the non-DKA group than in the control (*p* = 0.02 and 0.001 respectively) (Table 1). The platelet counts of the DKA and non-DKA groups were significantly higher than the control group (*p* < 0.001in both) (Table 1). At time of admission, the DKA group’s hydroxybutyrate in urine was elevated (9.6 ± 1.22 mmol/l) and their arterial pH and serum HCO3^-^ were low (7.07 ± 0.2 and 8.16 ± 4.3 mmol/l respectively), which are from DKA diagnostic criteria according to NICE guidelines 2015 [14].

**Table 1:** Demographic and laboratory characteristics of the studied children

Variable	DKA (*n* = 30)	Non-DKA (*n* = 30)	Controls (*n* = 30)	*p*-value		
				†p	‡p	§p
Age (years): mean ± SD	10.14 ± 3.29	11.84 ± 4.75	9.4 ± 2.8	0.09	0.05	0.07
Sex: Males: n (%)	10 (33.33%)	12 (40%)	12 (40%)	0.6	0.63	0.79
Females: n (%)	20 (66.67%)	18 (60%)	18 (60%)			
Plasma glucose (mmol/l): mean ± SD	28.4 ± 3.1	8.5 ± 2.1	6.1 ± 0.6	<0.001*	<0.001*	0.02*
Glycated HbA1c (%): mean ± SD	12.66 ± 1.9	6.63 ± 0.69	5.9 ± 0.5	<0.001*	<0.001*	<0.001*
Serum creatinine (mg/dl): mean ± SD	0.57 ± 0.21	0.59 ± 0.22	0.45 ± 0.22	0.86	0.13	0.12
BUN (mg/dl): mean ± SD	15.44 ± 3.1	13.9 ± 2.2	13.4 ± 2.1	0. 14	0.24	0.64
Platelet count (×10^9^/l): mean ± SD	260.2 ± 47.2	174.6 ± 17.9	142.9 ± 58.2	<0.001*	<0.001*	0.08
MPV(fl): mean ± SD	11.23 ± 0.8	10.6 ± 0.7	10.3 ± 0.7	<0.001*	<0.001*	0.061
PDW(fl): mean ± SD	14.7 ± 2.5	11.8 ± 1.7	11.2 ± 2.6	<0.001*	<0.001*	0.937
Serum BChE activity (U/l): mean ± SD	5078 ± 2008	7197 ± 2389	6296 ± 1989	0.003*	0.049	0.06

*BUN* blood urea nitrogen; *MPV* mean platelet volume; *PDW* platelet distribution width; *BChE* butyrylcholinesterase; *fl* femtoliter

†*p* = DKA vs non-DKA; ‡*p* = DKA vs control; §*p* = non-DKA vs. control

*statistical significance at p < 0.05

### Platelet parameters and serum BChE activity

We compared MPV, PDW and serum BChE activity levels in the DKA group to the other two groups (Table 1). We found that MPV and PDW were significantly higher (*p* < 0.001 in all); and serum BChE activity was significantly lower in the DKA group than the non-DKA and control groups (*p* = 0.04 and 0.003). But, there were no significant differences between the control and the non-DKA groups regarding MPV, PDW or serum BChE activity (*p* > 0.05).

**Fig. 1:**
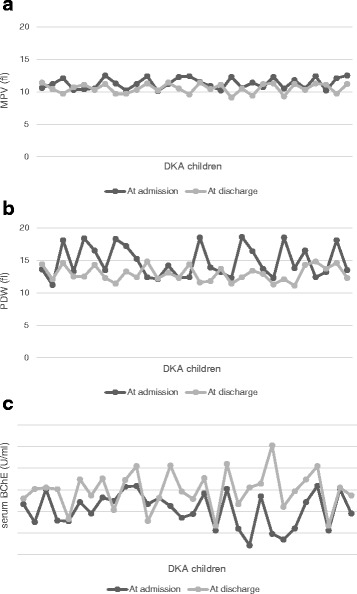
Platelet parameters and serum BChE activity in DKA group at admission and at discharge. (*n* = 30); 1**a** Mean platelet volume, 1**b** Platelet distribution width, 1**c** serum BChE activity, p <0.001 in all

Furthermore, by the time the DKA children were discharged from the hospital after resolution of DKA, MPV and PDW decreased significantly (Fig. 1a and b); and serum BChE activity increased significantly (Fig. 1c) (*p* < 0.001 in all) than their levels when the children were first admitted.

### ROC curve analysis and validity tests of platelet parameters and serum BChE activity

**Fig. 2:**
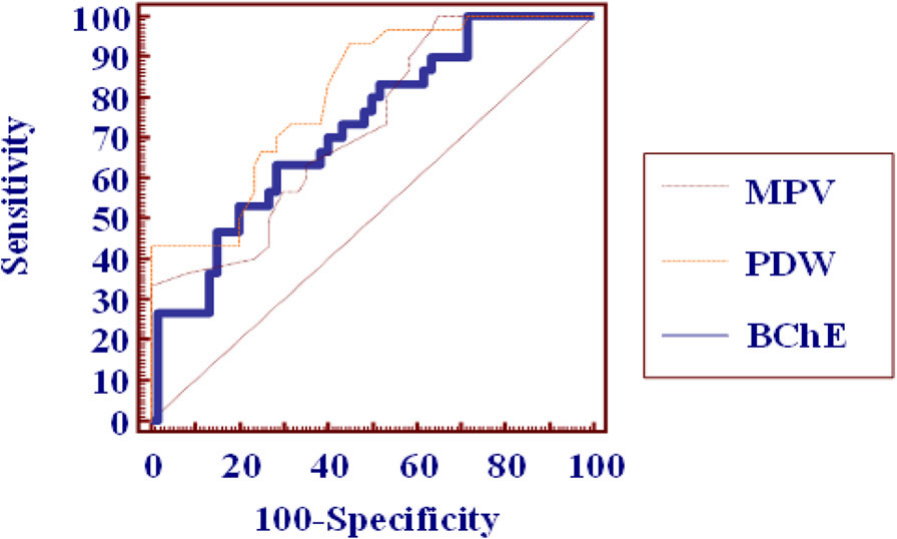
ROC curve for platelet parameters & serum BChE

Figure 2 shows ROC curve of platelet parameters and serum BChE activity, while their validity tests are shown in Table 2. We compared the sensitivity and specificity of the platelet parameters and serum BChE activity in predicting DKA, and there were no significant differences observed between BChE and MPV (z = 0.0557, *p* > 0.05), or MPV and PDW (z = 1.118, *p* > 0.05) or PDW and BChE (z =1.030, *p* > 0.05).

**Table 2:** Validity tests for platelet parameters and serum BChE activity regarding DKA prediction

Variable	AUC	Cut-off	PPV	NPV	Sensitivity	Specificity
MPV	0.713	>9.8 (fl)	56.7%	100%	100%	35%
PDW	0.798	>12.2 (fl)	63.2%	90.2%	93%	58%
Serum BChE activity	0.72	<5859 (U/l)	47.7%	32.9%	63.3%	71.7%

*MPV* mean platelet volume; *PDW* platelet distribution width; *BChE* butyrylcholinesterase; *fl* femtoliter; *AUC* area under curve; *PPV* positive predictive value; *NPV* negative predictive value

Multiple logistic regression analyses of platelet parameters and serum BChE as risk markers for DKA showed that odd Ratio and 95% confidence intervals (CIs) were significantly higher in MPV than PDW or BChE (OR = 4.251, CI 95% =1.463–12.351, *p* = 0.003; Table 3).

**Table 3:** Multiple logistic regression analyses of risk markers for DKA

Variable	OR	CI 95%	*p*-value
MPV(fl)	4.251	1.463–12.351	0.003*
PDW(fl)	2.265	1.358–3.778	0.002*
Serum BChE activity (U/l)	0.999	0.999–1.000	0.01*

*OR* Odd Ratio; *CI 95%* 95% confidence intervals; *MPV* mean platelet volume; *PDW* platelet distribution width; *BChE* butyrylcholinesterase; *fl* femtoliter

*statistical significance at *p* < 0.05

Regarding correlations in our study, we found only HbA1c to have significant correlations with MPV (*r* = 0.36, *p* = 0.002), PDW (*r* = 0.37, *p* = 0.001) and serum BChE (*r* =−0.91, *p* = 0.008), whereas, they did not correlate significantly with plasma glucose, arterial PH or serum HCO3^-^ (*p* > 0.05).

## Discussion

In our study, the platelet count, MPV, and PDW levels were significantly higher in the DKA group than the other two groups (*p* < 0.001). This can be attributed to the cytokines released during inflammatory conditions, like DKA. These cytokines influence the increase in megakaryocyte ploidy and volume; that allows megakaryocyte to produce several thousand platelets per cell [16]. Platelets produced are activated and are larger and more reactive [17], with wide variations in their size [18]. Platelet activation is likely to contribute to the known increased risk of diabetic complications. It is reported that soluble CD40 ligand and platelet surface bio-factors are correlated with diabetic nephropathy [19]. Recently, platelet indices, including MPV and PDW, were reported to be beneficial prognostic markers of diabetic retinopathy [20]. On the other hand, we noted that there were no significant differences between the non-DKA and the control groups regarding platelet count, MPV and PDW levels (*p* > 0.05). This contradicts with many studies that found them significantly higher in diabetic subjects [21–23]. They explained that inadequate glycemic control lead to protein glycation and oxidative stress; this causes platelet activation with altered platelet morphology and function [24]. However, platelet activity was found to recover with improved glycemic control [25], and our non DKA group may be considered as having good glycemic control (HbA1c = 6.63 ± 0.69%, 49 ± 7.5 mmol/mol).

Serum BChE activity was significantly lower in the DKA group than the other two groups (*p* < 0.05). Serum BChE activity is inhibited by oxidative stress and systemic inflammatory process generated in DKA; and this effect is reversed when inflammation subside [26]. Others suggested that serum BChE activity inhibition may be involved in reducing inflammation and oxidative stress in patients with ketoacidosis [21, 27]. In our study, no significant difference was found between the non-DKA and the control groups regarding serum BChE activity (*p* > 0.05), while a previous study reported a significant difference between them [21]. The insignificant difference in our results may be due to the regular insulin therapy and good glycemic control of the non DKA group which inhibited inflammation and reduced oxidative stress [28, 29].

After correction of DKA, we noticed a significant decrease in MPV and PDW, while serum BChE activity significantly increased (*p* < 0.001 in all). During DKA management, receiving insulin and fluid therapy abolishes the cascade of releasing inflammatory mediators and cytokines, which in turn inhibits platelet activation and increase serum BChE activity. Insulin therapy inhibits hyperglycemia, thus attenuates glucose-mediated inflammation, which suppresses pro-inflammatory cytokines and induces anti-inflammatory mediators [28]. Correction of dehydration and hypoperfusion by fluid therapy, reduces lactic acidosis which in turn decreases inflammatory mediators release [2].

We found that MPV at a cutoff value of > 9.8 fl was the most sensitive (100%) with highest NPV (100%), PDW at a cutoff value of >12.2 fl had the highest PPV (63.2%), and serum BChE activity at a cutoff value of < 5859 U/l was the most specific (71.7%). But these differences in the sensitivity and specificity among the three variables did not reach statistical significance (*p* > 0.05) when the optimum diagnostic cut-off values for the estimation of DKA was evaluated, so the three variables can equally predict the presence of DKA. This was in agreement with what Ma et al. (2013) found in their adult diabetic patients [21].

When we applied multiple logistic regression analysis, it revealed elevated MPV to be the most suitable risk marker for DKA. While Ma et al. (2013) found the most suitable risk marker to be the elevated PDW [21]. Many studies confirm the important role of MPV as a marker in several inflammatory disorders as diabetes mellitus, which confirms our result [30]. MPV is a relative reliable marker of thrombopoiesis and platelet function understanding in different thrombotic and inflammatory conditions [5], while the prognostic value PDW is still questionable as the variability of platelet size is not well explored [31].

Finally, MPV, PDW and serum BChE activity correlated significantly only with HbA1c (*p* < 0.05). This can be attributed to the fact that chronic hyperglycemia influences inflammatory mediator which inhibit the activity of serum BChE [28]. Moreover, chronic hyperglycemia increases platelet activity by inducing non-enzymatic glycation of proteins on the surface of the platelet; by the osmotic effect of glucose and activation of protein kinase C [32].

However, platelet parameters and serum BChE activity had no significant correlations with DKA severity (arterial PH and serum HCO3 [14]) (*p* > 0.05 in all). Many experimental studies suggest that strong acidification is a necessity to show the link of acidosis with inflammation, and these levels of acidosis are rarely observed in clinical situations [33].

## Conclusion

From our results we can conclude that platelet parameters and serum BChE activity change significantly when children develop DKA, while they have no role on predicting the severity of it. We found MPV to be the most suitable marker for clinical monitoring of DKA. We advise clinicians to pay more attention to the platelet parameters and serum BChE activity when diabetic children develop DKA. As far as we know this is the first study to assess platelet parameters and serum BChE activity in diabetic children. Our study has several limitations, for example, larger population based studies may find statistically significant differences among the mild, moderate, and severe DKA groups and further studies are needed to know if MPV, PDW and serum BChE are related to complications of diabetes other than DKA.

## Abbreviations

BChE: Butyrylcholinesterase
HbA1c: Glycated hemoglobin
MPV: Mean platelet volume
PDW: Platelet distribution width

## References

[1] Cooke DW, Plotnick L (2008). Management of diabetic ketoacidosis in children and adolescents. Pediatr Rev.

[2] Wolfsdorf JI, Allgrove J, Craig ME, Edge J, Glaser N, Jain V, Lee WWR, Mungai LNW, Rosenbloom AL, Sperling MA, Hanas R (2014). A consensus statement from the international society for pediatric and adolescent diabetes: diabetic ketoacidosis and hyperglycemic hyperosmolar state. Pediatr Diabetes.

[3] Karavanaki K, Karanika E, Georga S, Bartzeliotou A, Tsouvalas M, Konstantopoulos I, Fotinou A, Papassotiriou I, Karayianni C (2011). Cytokine response to diabetic ketoacidosis (DKA) in children with type 1 diabetes (T1DM). Endocr J.

[4] Hoffman WH, Stamatovic SM, Andjelkovic AV (2009). Inflammatory mediators and blood brain barrier disruption in fatal brain edema of diabetic ketoacidosis. Brain Res.

[5] Gasparyan AY, Ayvazyan L, Mikhailidis DP, Kitas GD (2011). Mean platelet volume: a link between thrombosis and inflammation?. Curr Pharm Des.

[6] Boos CJ, Lip GY (2007). Assessment of mean platelet volume in coronary artery disease - what does it mean?. Thromb Res.

[7] Wiwanitkit V (2004). Plateletcrit, mean platelet volume, platelet distribution width: its expected values and correlation with parallel red blood cell parameters. Clin Appl Thromb Hemost.

[8] Miller RD (2005). Miller’s Anesthesia.

[9] Mourad TA (2005). Adverse impact of insecticidal on the health of Palestinian farm workers in the Gaza strip: A haematologic biomarker study. Int J Occup Environ Health.

[10] Das UN (2012). Acetylcholinesterase and butyrylcholinesterase as markers of low-grade systemic inflammation. Ann Hepatol.

[11] Omu AE, Al-Azemi MK, Omu FE, Fatinikun T, Abraham S, George S, Mahnazhath N (2010). Butyrylcholinesterase activity in women with diabetes mellitus in pregnancy: Correlation with antioxidant activity. J Obstet Gynaecol.

[12] Nelson LS, Ford MD, Goldman L, Schafer AI (2016). Acute poisoning. Goldman’s Cecil Medicine.

[13] Stojanov M, Stefanovic A, Dzingalasevic G, Mandic-Radic S, Prostran M (2011). Butyrylcholinesterase activity in young men and women: Association with cardiovascular risk factors. Clin Biochem.

[14] National Institute for Health and Care Excellence (NICE). Diabetes (type 1 and type 2) in children and young people: diagnosis and management. NICE guidelines [NG18]. Published date: August 2015. http://www.nice.org.uk/guidance/ng18.

[15] American Diabetes Association (2010). Diagnosis and classification of diabetes mellitus. Diabetes Care.

[16] Mattia G, Vulcano F, Milazzo L, Barca A, Macioce G, Giampaolo A, Hassan HJ (2002). Different ploidy levels of megakaryocytes generated from peripheral or cord blood CD34+ cells are correlated with different levels of platelet release. Blood.

[17] Oncel MY, Ozdemir R, Yurttutan S, Canpolat FE, Erdeve O, Oguz SS, Uras N, Dilmen U (2012). Mean platelet volume in neonatal sepsis. J Clin Lab Anal.

[18] Artunc Ulkumen B, Pala HG, Calik E, Oruc KS (2014). Platelet distribution width (PDW): A putative marker for threatened preterm labour. Pak J Med Sci.

[19] Lajer M, Tarnow I, Michelson AD, Jorsal A, Frelinger AL, Parving HH, Rossing P, Tarnow L (2010). Soluble CD40 ligand is elevated in type 1 diabetic nephropathy but not predictive of mortality, cardiovascular events or kidney function. Platelets.

[20] Yilmaz T, Yilmaz A. Relationship between Altered Platelet Morphological Parameters and Retinopathy in Patients with Type 2 Diabetes Mellitus. Journal of Ophthalmology. vol. 2016, Article ID 9213623, 5 pages, 2016. doi:10.1155/2016/9213623 10.1155/2016/9213623 PMC4844893 27190641

[21] Ma SG, Yang LX, Qiu XQ (2013). Assessment of the platelet parameters and serum butyrylcholinesterase activity in type 1 diabetes patients with ketoacidosis. Platelets.

[22] Jindal S, Gupta S, Gupta R, Kakkar A, Singh HV, Gupta K, Singh S (2011). Platelet indices in diabetes mellitus: Indicators of diabetic microvascular complications. Hematology.

[23] Malachowska B, Tomasik B, Szadkowska A, Baranowska-Jazwiecka A, Wegner O, Mlynarski W, Fendler W (2015). Altered Platelets’ morphological parameters in children with type 1 diabetes – a case-control study. BMC Endocr Disord.

[24] Jabeen F, Fawwad A, Rizvi HA, Alvi F (2013). Role of platelet indices, glycemic control and hs-CRP in pathogenesis of vascular complications in type-2 diabetic patients. Pak J Med Sci.

[25] Demirtunc R, Duman D, Basar M, Bilgi M, Teomete M, GDemirtunc R, Duman D, Basar M, Bilgi M, Teomete M, Garip T.arip T. The relationship between glycemic control and platelet activity in type 2 diabetes mellitus. J Diabetes Complications. 2009;23(2):89-94. 10.1016/j.jdiacomp.2008.01.006 18358749

[26] Hubbard RE, O’Mahony MS, Calver BL, Woodhouse KW (2008). Plasma esterases and inflammation in ageing and frailty. Eur J Clin Pharmacol.

[27] Erbagci AB, Tarakcioglu M, Coskun Y, Sivasli E, Sibel NE (2001). Mediators of inflammation in children with type I diabetes mellitus: Cytokines in type I diabetic children. Clin Biochem.

[28] Sun Q, Li J, Gao F (2014). New insights into insulin: The anti-inflammatory effect and its clinical relevance. World J Diabetes.

[29] Tagliari B, dos Santos TM, Cunha AA, Lima DD, Delwing D, Sitta A, Vargas CR, Dalmaz C, Wyse AT (2010). Chronic variable stress induces oxidative stress and decreases butyrylcholinesterase activity in blood of rats. J Neural Transm (Vienna).

[30] Karaman H, Karakukcu C, Kocer D (2013). Can mean platelet volume serve as a marker for prostatitis?. Int J Med Sci.

[31] Cooke J, Murphy T, McFadden E, O’Reilly M, Cahill MR (2009). Can mean platelet component be used as an index of platelet activity in stable coronary artery disease?. Hematology.

[32] Kakouros N, Rade JJ, Kourliouros A, Resar JR (2011). Platelet function in patients with diabetes mellitus: from a theoretical to a practical perspective. Int J Endocrinol.

[33] de Nadai TR, de Nadai MN, Albuquerque AA, de Carvalho MT, Celotto AC, Evora PR (2013). Metabolic acidosis treatment as part of a strategy to curb inflammation. Int J Inflam.

